# Physiological and growth responses to water deficit in the bioenergy crop *Miscanthus x giganteus*

**DOI:** 10.3389/fpls.2013.00468

**Published:** 2013-11-25

**Authors:** Jennifer Ings, Luis A. J. Mur, Paul R. H. Robson, Maurice Bosch

**Affiliations:** Institute of Biological, Environmental and Rural Sciences, Aberystwyth UniversityAberystwyth, UK

**Keywords:** *Miscanthus*, drought, water deficit, physiology, metabolite profiling, stress, bioenergy

## Abstract

High yielding perennial biomass crops of the species *Miscanthus* are widely recognized as one of the most promising lignocellulosic feedstocks for the production of bioenergy and bioproducts. *Miscanthus* is a C_4_ grass and thus has relatively high water use efficiency. Cultivated *Miscanthus* comprises primarily of a single clone, *Miscanthus* x *giganteus*, a sterile hybrid between *M. sacchariflorus* and *M. sinensis*. *M.* x *giganteus* is high yielding and expresses desirable combinations of many traits present in the two parental species types; however, it responds poorly to low water availability. To identify the physiological basis of the response to water stress in *M.* x *giganteus* and to identify potential targets for breeding improvements we characterized the physiological responses to water-deficit stress in a pot experiment. The experiment has provided valuable insights into the temporal aspects of drought-induced responses of *M.* x *giganteus*. Withholding water resulted in marked changes in plant physiology with growth-associated traits among the first affected, the most rapid response being a decline in the rate of stem elongation. A reduction in photosynthetic performance was among the second set of changes observed; indicated by a decrease in stomatal conductance followed by decreases in chlorophyll fluorescence and chlorophyll content. Measures reflecting the plant water status were among the last affected by the drought treatment. Metabolite analysis indicated that proline was a drought stress marker in *M.* x *giganteus*, metabolites in the proline synthesis pathway were more abundant when stomatal conductance decreased and dry weight accumulation ceased. The outcomes of this study in terms of drought-induced physiological changes, accompanied by a proof-of-concept metabolomics investigation, provide a platform for identifying targets for improved drought-tolerance of the *Miscanthus* bioenergy crop.

## INTRODUCTION

Decreasing water availability, as a result of climate change, will lead to prolonged dry periods and hence reduced availability or increased variability in water resources in mid-latitudes and semi-arid low latitudes (IPCC, 2007). This combined with an increasing population and increasing societal water demands will lead to water resources being a scarce commodity for agricultural purposes (Rosegrant and Cline, 2003). Drought or water deficit affects crop yield more than any other environmental stress worldwide (Cattivelli et al., 2008), negatively impacting on plant growth, development, survival, and crop productivity, posing a substantial threat to sustainable agriculture (Boyer, 1982).

Biomass from dedicated high yielding bioenergy crops, including tropical C_4_ grasses from the genus *Miscanthus*, has been identified as a major source for the production of renewable energy (Carroll and Somerville, 2009; Feltus and Vandenbrink, 2012). Hence, drought induced decreases in yield are of major concern for the development of *Miscanthus* cultivars that are sustainable and economically viable biomass feedstocks.

*Miscanthus* is a woody, perennial rhizomatous grass, with a wide indigenous geographical distribution in East-Asia and the genotypes arising from these varying climates differ in their optimal growth condition. While a lot of the research and breeding focus is on the development of *Miscanthus* hybrids and varieties with improved lignocellulosic biomass yield and conversion efficiencies, the development of drought-tolerant lines will become increasingly important as water resources become more limiting.

Despite water use efficiency of C_4_ crops often being higher than that of C_3_ crops (Long, 1999; Gowik and Westhoff, 2011), water availability still dictates the maximum yields achievable by a C_4_ crop such as *Miscanthus*. The most widely grown and best studied *Miscanthus* species so far is *Miscanthus* x *giganteus*, a sterile hybrid of *M. sacchariflorus* and *M. sinensis* parentage (Hodkinson et al., 2002). *M.* x *giganteus*, also referred to as Asian elephant grass, probably has the greatest biomass potential to date with reported dry matter yields after complete plant senescence of 4–32 t ha^-^^1^ year^-^^1^ in Europe with higher yields in Southern Europe (Lewandowski et al., 2000). Growth trials in the US state of Illinois showed an average yield of 30 t ha^-^^1^ year^-^^1^ with a significantly higher productivity than maize (*Zea mays*) and switchgrass (*Panicum virgatum*) in side-by-side trials (Heaton et al., 2008; Dohleman and Long, 2009). Stabilizing crop performance under drought, which in effect means increasing crop productivity per unit of applied water, will be a main priority for *Miscanthus* in particular when it is to be grown on marginal land, with little irrigation.

It has been shown that plants perceive and respond rapidly to even small alterations in water status via physiological, cellular, and molecular events. These responses are determined by the intensity, duration, and rate of progression of the water stress (Chaves et al., 2003). The different physiological changes that can be induced upon drought are well documented. However, the type and timing of physiological responses to drought can vary in different species and between genotypes (Merchant et al., 2007; Centritto et al., 2009; Costa et al., 2012).

While it is clear that unimproved *M.* x *giganteus* possesses a range of agronomically desirable traits as a bioenergy feedstock, studies have shown it to be less drought tolerant compared to its parent species, in particular *M. sinensis* (Clifton-Brown and Lewandowski, 2000) and that drought stress negatively impacts on its yield (Price et al., 2004; Maughan et al., 2012). Despite this, little is known about the physiological traits associated with drought stress in *M.* x *giganteus*.

The main objective of this study was to characterize the physiological responses, and the timing of these responses that *M.* x *giganteus* undergoes when exposed to water stress. This knowledge is important especially considering that bioenergy crops like *M.* x *giganteus* are expected to generate high yields on less productive soils with minimal irrigation. Mapping the physiological changes in *M.* x *giganteus* upon drought stress will improve our capacity to evaluate and predict the agronomic performance of this energy crop in response to extreme environments.

Drought elicits substantial changes in plant metabolism as plants accumulate compatible osmolytes inside the plant cell to retain water and maintain positive turgor pressure (Verslues and Juenger, 2011). In addition to relevant phenotype data under water stress we present data showing associated changes in overall metabolite profiles.

The outcomes of this study provide a platform for the identification of potential targets for breeding improvements of the *Miscanthus* bioenergy crop.

## MATERIALS AND METHODS

### PLANT MATERIAL

*M.* x *giganteus* rhizomes were collected in April 2012 from plants grown as part of a field trial in Aberystwyth, UK. After brief storage at 4°C, 35 rhizomes with a weight of 20 ± 5 g were planted in individual 25 cm diameter pots containing John Innes No. 3 commercial potting compost. The pots were placed in a glasshouse at 24°C with 18 h of light, and initial growth rate of plants recorded during May–June 2012.

### EXPERIMENTAL DESIGN

The plants were split into five groups of seven replicates with equal standard deviations of height after 2 months of growth. These were placed in a completely randomized design and incubated under the same greenhouse conditions as above. All plants within the five groups were initially watered every 2 days with water being withheld from the water-stressed plants (two groups) from day 12. Selected plants were destructively harvested on day 12 (one group: T0), 24 (two groups: control 1, C1; drought 1, D1), and 32 (two groups: control 2, C2; drought 2, D2). Non-destructive measurements were performed on all plants including those to be removed at destructive harvests on day12, 24, and 32.

### PHYSIOLOGICAL MEASUREMENTS

All measurements were made every 2 days between 22 June–24 July 2012 and were taken from equivalent leaves and from the tallest stem (at beginning of experiment) where multiple stems were present.

Soil moisture content was recorded using a hand-held moisture sensor (SM300 and HH2 moisture meter, Delta-T Devices Ltd., Cambridge, UK), taking the average of three measurements from each pot.

Stomatal conductance was measured between 12:00 and 14:00 h on the youngest leaf with a fully expanded ligule (leaf 0) using an AP4 porometer (Delta-T devices Ltd, Cambridge, UK).

Chlorophyll fluorescence was measured between 10:30 and 12:00 h on three leaves per plant [leaf 0, -2 (two leaves older than leaf 0), and 2 (second youngest leaf after leaf 0)] with a Handy PEA continuous excitation chlorophyll fluorimeter (Hansatech Instruments Ltd., Norfolk, UK). When using the PEA, the attached leaf was dark-adapted with a leaf clip for 30 min before the measurement. During the measurement the PEA sensor unit was held over the clip and the shutter opened. A high intensity LED array on the sensor head provided a maximum light intensity of 3000 μmol m^-^^2^ s^-^^1^, sufficient to ensure closure of all PSII reaction centers. Maximal PSII photochemical efficiency *F*_v_*/F*_m_ (ratio of variable fluorescence to maximum fluorescence) was calculated automatically and recorded. The high data acquisition of 10 μs for the first 2 ms allowed rapid chlorophyll a transients to be determined from the polyphasic curve which were used to calculate additional parameters including performance index (PI) and the quantum yield of electron transport (Oukarroum et al., 2007).

Chlorophyll content was measured on five leaves (-2, -1, 0, 1, 2; denomination as above) between 10:00 and 12:00 h using a SPAD-502 m (Konica Minolta Optics Inc.). Three readings were taken at quarterly intervals along the leaf and the mean of the values recorded.

Relative water content (RWC) was measured on day 12, 24, and 32, using samples taken from two leaves per plant (leaf -1 and 1). The RWC was calculated as follows and means were calculated for each plant and treatment:

RWC (%)=[(FW–DW)/(TW–DW)]×100

(where: FW = fresh weight, DW = dry weight, and TW = turgid weight)

Fresh weight was determined at time of cutting, turgid weight after 24 h in sterile distilled water and dry weight after 72 h drying in a 60°C oven.

Plant water content was evaluated from total above ground biomass measurements taken on day 12, 24, and 32. Fresh weight was recorded at harvest and dry weight was the constant weight achieved after drying in a 60°C oven. Water content was calculated on a dry weight basis as follows:

WC⁢(g/g)=(F⁢W⁢–⁢D⁢W)/DW

### GROWTH MEASUREMENTS

Stem elongation was measured every 2 days from soil level to the highest fully expanded ligule (leaf 0) using a graduated ruler. The rate of elongation was then calculated using these measurements.

Leaf expansion was measured on leaf 0 with leaf length (from the ligule to leaf tip) and width (midway between ligule and tip) measured using a graduated ruler. Leaf area was calculated as described (Clifton-Brown and Lewandowski, 2000):

$$Area\,\left(c\,m^{2}\right)=0.74\times length\;\left(c\,m\right)\times width\,\left(cm\right)$$

The rate of expansion was calculated using the leaf area values.

### METABOLIC ANALYSIS

Leaf samples were prepared using ground tissue from leaf 0 and the extraction procedure followed that of Allwood et al. (2006). Metabolites were analyzed using Direct Injection Electrospray Ionization Mass Spectrometry (DI-ESI-MS) on a Micromass LCT mass spectrometer (Micromass/Waters Ltd., UK) in negative ionization mode where metabolites are singly ionized by the loss of H^+^. The polar extracts were reconstituted in 0.25 mL 30 % [v/v] methanol : H_2_O and 50 μL added to 200 μL inserts in 2 mL (Waters Ltd. UK) and introduced by direct-infusion at a flow rate of 0.05 mL min^-^^1^ in 30 % [v/v] methanol : H_2_O running solvent. Data were acquired over the *m/z* range 100–1400 Th and were imported into MATLAB (The MathWorks Inc., Natwick, MA, USA), binned to unit mass and then normalized to percentage total ion count as described in Johnson et al. (2007).

### STATISTICAL ANALYSIS

Measurements were performed on all remaining plants, minimum seven plants per treatment at each time point, and a mean value calculated for each treatment at each time point. All values are expressed as mean ± SEM. All analyses were performed using Minitab version 14 (Minitab Inc., Coventry, UK). Statistical differences were estimated from ANOVA tests at the 5% level (*p* ≤ 0.05) of significance, for all parameters evaluated. Where ANOVA indicated a significant difference, a pair-wise comparison of means by Fisher’s least significant difference (LSD) was carried out. Regression was used to fit lines to the data.

Metabolite data were analyzed using principal components analysis (PCA) following accepted Metabolomics Standard Initiative procedures (Sansone et al., 2007). PCA is an unsupervised method where no *a priori* knowledge of experimental structure is given. Thus, if there is clustering of either 2D or projections of PCA from replicate data, this indicates that the original experimental parameters are the sources of maximal variation.

## RESULTS

### SOIL MOISTURE CONTENT AND RELATIVE WATER CONTENT

**FIGURE 1:**
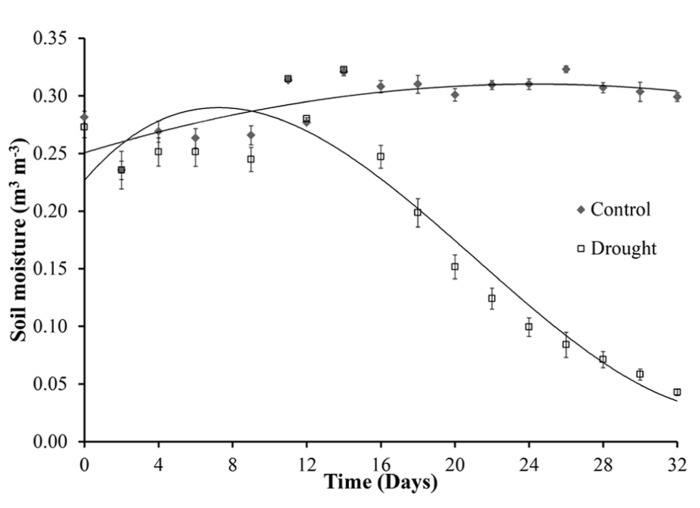
**Soil moisture.** Soil moisture content was measured every 2 days over a period of 32 days. The last watering of the drought stressed plants was on day 12. The control plants continued to be watered every 2 days for the duration of the experiment. A significant decline in soil moisture occurred on day 16, 4 days after final watering, with a steady decline over the remaining period to levels similar to drought in grassland ecosystems.

**Figure 1** shows the variation of soil moisture content during the experiment. The final watering of the drought stressed plants was on day 12. From day 16 the volumetric soil moisture content decreased significantly (*p* < 0.001) in water stressed plants when compared with the watered control plants that maintained a constant soil moisture content of 0.3 m^3^ m^-^^3^. During the course of the drought experiment the soil moisture content readings decreased to 0.05 m^3^ m^-^^3^, similar levels of soil moisture were observed during natural drought in a grassland ecosystem (Mikkelsen et al., 2008).

**FIGURE 2:**
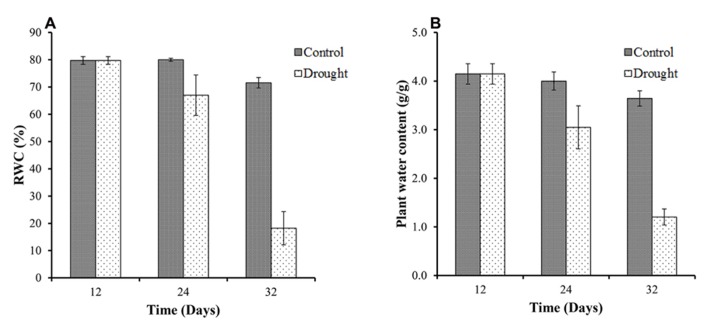
**Leaf relative water content and above ground biomass moisture content.** The control plants maintained a constant leaf RWC of between 72 and 80% throughout the duration of the investigation **(A)**. In water-stressed plants leaf RWC declined from 80% at the start of the investigation to <20% by day 32. Total above ground biomass moisture content followed a very similar pattern to the leaf RWC **(B)**.

Relative water content measurements determine plant water status at destructive harvests. All plants showed high values of leaf RWC in well-watered conditions at the beginning of the study with an average RWC of 80% at day 12 (**Figure 2A**). The effect of the water stress was evident at day 24, 12 days after water withdrawal, with a decrease from 80% leaf RWC in control plants to <70% in the water stressed plants. By day 32 there was a significant (*p* < 0.001) treatment difference for leaf RWC between the two groups with the water stressed group declining to <20% leaf RWC. As expected, the total above ground biomass moisture content (**Figure 2B**) followed a very similar pattern to the leaf RWC.

**FIGURE 3:**
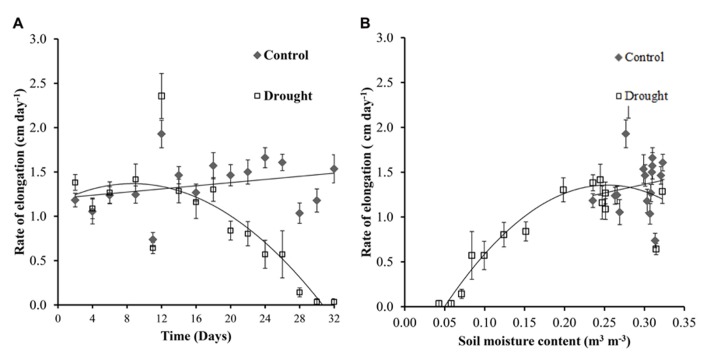
**Stem elongation.** Control plants showed a fairly constant rate of stem elongation throughout the investigation **(A)**. Water-stressed plants showed a significant decrease in elongation rate from day 20 **(A)** when the soil moisture decreased to <0.2 m^3^ m^-^^3^ **(B)**.

The rate of stem elongation remained fairly constant in the well-watered plants, with fluctuations in growth rate most likely caused by changing identities of the uppermost leaf with a fully expanded ligule (leaf 0). Stem elongation rates in well-watered and water-stressed *Miscanthus* diverged significantly (*p* = 0.01) at day 20 (**Figure 3A**) which corresponds to a soil moisture content of below 0.2 m^3^ m^-^^3^ in the water-stressed plants (**Figure 3B**). Elongation ceased completely at a soil moisture content of <0.05 m^3^ m^-^^3^.

**FIGURE 4:**
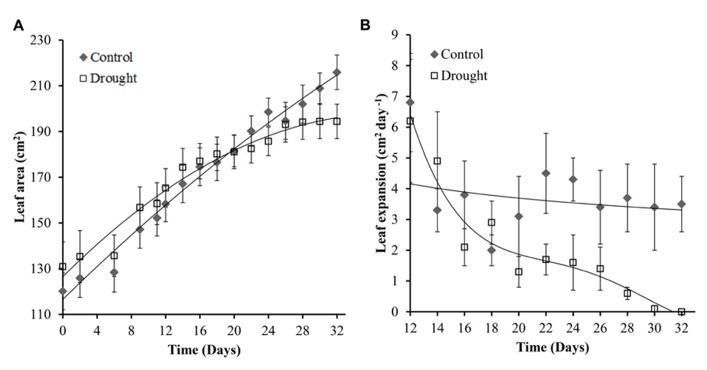
**Leaf area and expansion of leaf 0.** Leaf area showed a linear increase in control plants throughout the experiment while no significant increase after day 26 was observed in water-stressed plants **(A)**. Rate of leaf expansion remained constant in control plants and decreased gradually in drought treatment plants **(B)**. The leaf expansion rate became significantly different between the two treatments at day 28.

The general effect of mild drought on leaves is a reduction in leaf number (data not shown), rate of expansion and final leaf size. The rate of leaf expansion in well-watered plants was constant throughout the experiment; and decreased in water-stressed plants toward the end of the experiment (**Figure 4B**). Leaf area in water-stressed plants did not significantly increase after day 26 (**Figure 4A**) and rate of expansion was significantly different (*p* = 0.047) between the two treatment groups at day 28 (**Figure 4B**).

**FIGURE 5:**
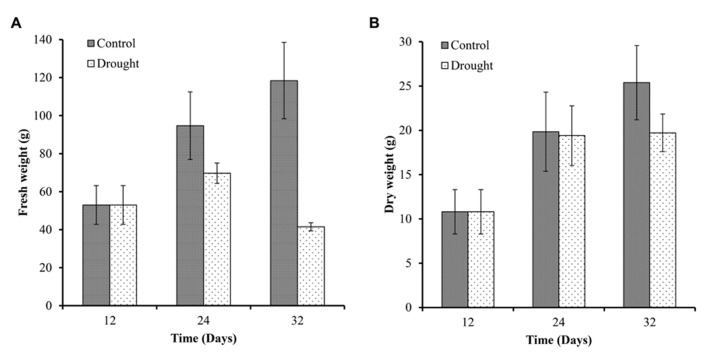
**Above ground biomass.** Fresh **(A)** and dry weights **(B)** of the sampled plants were measured at each harvesting point (*n* = 7). Fresh and dry biomass of control plants increased throughout the experiment. Water-stressed plants only showed a small increase in fresh weight between day 12 and 24 and a significant decrease at day 32. Dry weight accumulation was similar between the two treatments up to day 24 but dry biomass did not increase further in stressed plants between day 24 and 32.

To determine the effect of drought on plant harvestable yield, we measured the fresh weight and dry weight of the above ground biomass at three time points (**Figure 5**). Biomass increased in control plants throughout the investigation with a 79% increase in fresh biomass over 12 days between day 12 and 24 (**Figure 5A**) and an 84% increase in dry biomass (**Figure 5B**) during the same time period. Biomass increased a further 25% (fresh biomass) and 30% (dry biomass) during the following 8 days (**Figures 5A,B**). In water-stressed plants fresh biomass increased 31.5% over 12 days between day 12 and 24, and decreased by 40% between day 24 and 32. Biomass dry weight increased in water-stressed plants between day 12 and 24 to a similar extent as in well-watered plants and no further increase in biomass was measured in drought-stressed plants (**Figure 5B**).

### PHOTOSYNTHETIC PRODUCTIVITY

**FIGURE 6:**
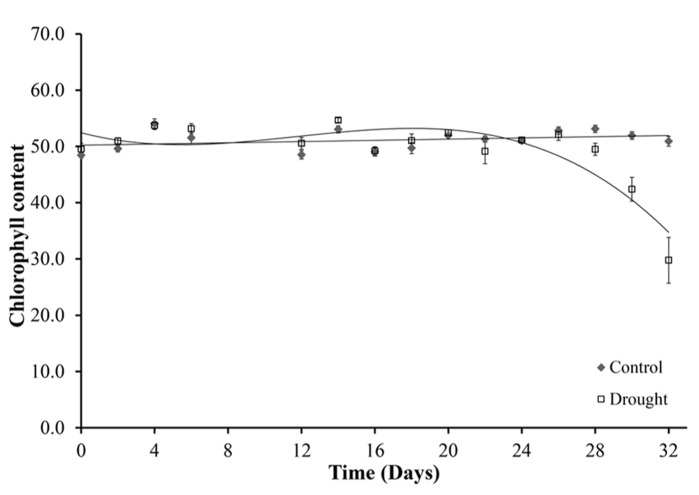
**Chlorophyll content.** Chlorophyll content was measured in five leaves per plant using a SPAD meter. Leaf 0 is shown because no significant differences were seen between the different leaves. Chlorophyll content was maintained in the well-watered plants for the duration of the experiment and decreased from day 28 under water-stressed conditions.

The chlorophyll content of leaves declined under drought stress (**Figure 6**). Chlorophyll content was determined for five leaves per plant, with no significant differences between the five leaves. The leaf chlorophyll content was constant in well-watered plants throughout the duration of the experiment (**Figure 6**). Water-stressed plants maintained chlorophyll levels until day 28 when a uniform significant decline in chlorophyll content occurred across all leaves (*p* < 0.02). At day 32 there was a 42% decline in leaf chlorophyll content of the water-stressed plants compared to the control well-watered plants.

**FIGURE 7:**
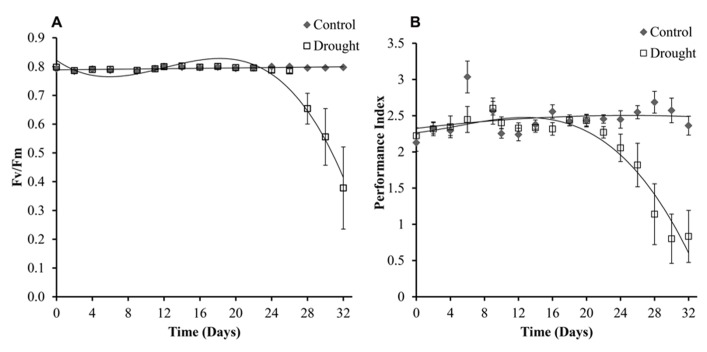
**Chlorophyll fluorescence.** The maximum quantum yield (*F*_v_/*F*_m_) **(A)** was maintained in well-watered plants for the duration of the experiment****and****decreased significantly in water-stressed plants from day 28. *F*_v_/*F*_m_ was measured in three leaves per plant and no significant differences were seen between the different leaves. Shown here is leaf 0. Performance index **(B)**, which incorporates more parameters than (*F*_v_/*F*_m_), was compared across the two treatments and was slightly more sensitive to drought differing significantly between the two treatments at day 26.

Chlorophyll fluorescence is a widely used method to research photosynthetic efficiency (Genty et al., 1989; Strasser et al., 1995) and was determined in three leaves per plant, with no significant differences being found between the leaves (*p* < 0.05). Levels of chlorophyll fluorescence were maintained at 0.8 *F*_v_/*F*_m_ in all leaves under controlled conditions and decreased under drought conditions (**Figure 7A**). The treatment groups became significantly different at day 28 (*p* < 0.04) across all different numbered leaves measured. Calculated PI was slightly more sensitive to drought treatment, and treatment groups were significantly different after 26 days (**Figure 7B**). To test if electron transport prior to the primary plastoquinone (Qa) determined drought induced changes in PI the correlation between log(PI) and log(φ o) (φo is the quantum yield of electron transport) was tested as described in Oukarroum et al. (2007) and was found to be linear (data not shown).

**FIGURE 8:**
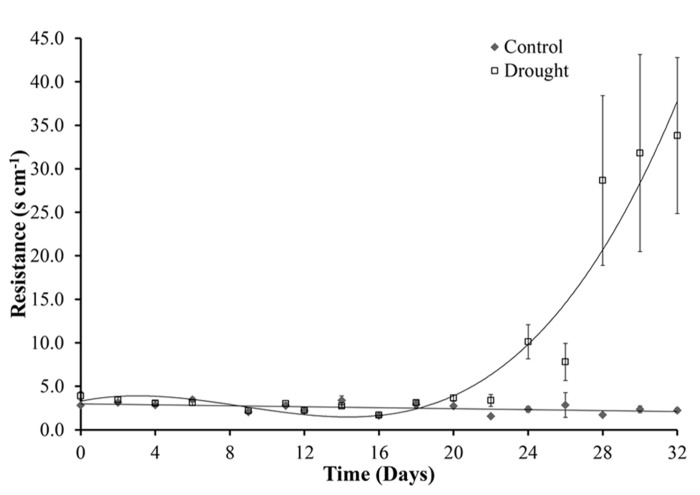
**Stomatal conductance.** Under controlled well-watered conditions stomatal conductance remained constant throughout the experiment. Water stress caused increased stomatal resistance and therefore a decrease in stomatal conductance, this became significantly different between the two treatments at day 24.

After cessation of watering, stomatal conductance was unchanged for 10 days (day 22). A significant difference (*p* = 0.002) between the two treatment groups was first observed at day 24. A rapid increase in resistance, corresponding to a decrease in stomatal size, from day 28 then followed until the end of the experiment at day 32 (**Figure 8**). The decrease in stomatal conductance preceded change in leaf area of the whole plant both in terms of new growth (leaf expansion **Figure 4**) and senescence of older leaves (chlorophyll content **Figure 6**).

### METABOLOMICS

**FIGURE 9:**
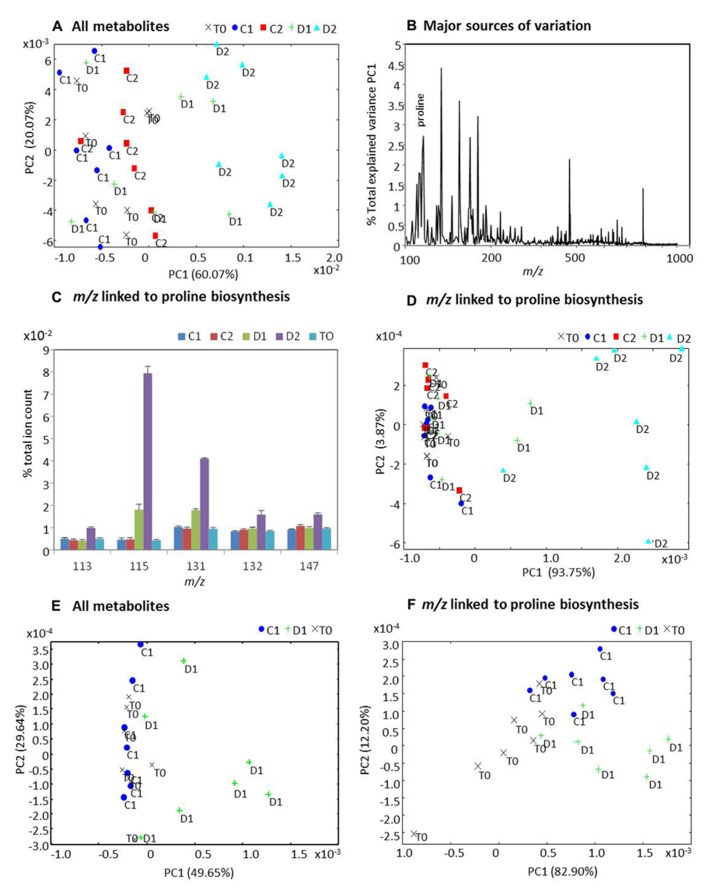
**Principal components analysis (PCA) of metabolite profiles from well-watered and water-stressed *Miscanthus* plants.** Polar and non-polar extracts from leaf samples of *Miscanthus* plants at the start of the experiment day 12 (T0) and from well-watered control plants at day 24 (C1) and day 32 (C2) and also from water-stressed plants at day 24 (D1) and day 32 (D2) were analyzed using direct infusion electrospray ionization mass-spectrometry (DI-ESI-MS). Derived spectra were analyzed by PCA both including C2, D2 **(A, D)** and excluding C2, D2 **(E, F)**. PCA plots **(A)** and **(D)** are based on data for all metabolites in the spectrum. PCA plots **(E)** and **(F)** are based on *m/z *within the spectra which were tentatively linked to metabolites forming the proline biosynthetic pathway namely L-glutamate (147 *m/z*);**L-ornithine (132 *m/z*); L-glutamate-semi-aldehyde (131 *m/z*), (*S*)-pyrroline-5-carboxylate (113 *m/z*), and L*-*proline (115 *m/z*). **(B)** The percentage contribution by each *m/z* to the variation explained by PC1 in **(A)**. **(C)** Percentage to total ion count contributed by each of the five metabolites tentatively associated with proline biosynthesis.

The polar/non-polar extracts from the samples were assessed using direct infusion electrospray ionization mass-spectrometry (DI-ESI-MS) and the derived spectra analyzed using PCA (**Figure 9**). PCA of derived spectra from all metabolites extracted across three time points and two treatments indicated that metabolites from the D2 group were distinctive to all other samples (**Figure 9A**). Analysis of the loadings showed that proline ranked amongst the major sources of variation (**Figure 9B**) contributing 2.78% to the total variation explained by PC1. Thus, *m/z* tentatively linked to the proline biosynthetic pathway were extracted from the spectra and all exhibited increased accumulation in the D2 group whilst *m/z* 115 (L-proline) and *m/z* 131 (L-glutamate-γ-semialdehyde) also increased in the D1 group **Figure 9C**). Separate analysis of *m/z* linked to proline biosynthesis by PCA again displayed clear separation of the D2 samples, further suggesting a significant contribution by this pathway to the *Miscanthus* drought response (**Figure 9D**). However, other analyses had suggested that *Miscanthus* plants at day 32 (20 days after cessation of watering) were under severe stress (**Figures 6** and **7**). Thus, the metabolomic data were re-analyzed with D2 and C2 samples excluded to highlight early responses which could be associated with drought tolerance mechanisms. The resulting PCA suggests that the metabolite profiles for some D1 samples were distinctive from the controls but others were not (**Figure 9E**). Similarly, when analyzing *m/z* linked to proline biosynthetic metabolites some D1 separation was observed but not for all samples (**Figure 9F**).

## DISCUSSION

Drought or water deficit is one of the most important factors affecting plant growth, development, survival, and crop productivity, posing a substantial threat to sustainable agriculture. Even the most productive agricultural regions experience short periods of drought and occasional severe drought periods. This has recently become more of an issue due to concerns over the effects of climate change on global agricultural productivity and hence food security and the socio-economic impacts associated with this. Although predictability of precipitation is uncertain, there seems to be a consensus among climate models showing that agricultural areas will be exposed to increasing periods of drought conditions (Falloon and Betts, 2010; Gornall et al., 2010; Trnka et al., 2011). As water resources become more limiting, the development of drought-tolerant crops will become increasingly important.

However, studies that seek to ameliorate the negative impact of drought on agricultural productivity have been mostly focussed upon annually harvested food crops. Many of these studies highlight the particular stages of development that are highly susceptible to drought. For grain crops drought is particularly impactful on crop yield if the water stress coincides with the period of grain filling because the harvest index is largely dependent on assimilate partitioning into grain. As a consequence the mechanisms to improve yield under drought in food crops include for example either avoidance of drought during grain filling such as early crop growth (Fischer et al., 1998; Araus et al., 2002; Farooq et al., 2011), or drought tolerance during grain filling such as stay-green phenotypes (Thomas and Howarth, 2000; Harris et al., 2007). *Miscanthus* is an undomesticated new crop, with several characteristics that distinguish it from many other crops previously studied in drought research. It is a high yielding crop in which all above ground biomass is harvested annually. This distinguishes it from other biomass crops, such as trees, in which biomass is harvested after several years growth. Therefore *Miscanthus* represents a mix of annual harvested yield and a perennial growth habit. Because of this unusual combination it is appropriate to consider the particular sensitivities that affect *Miscanthus* growth and that potentially impact upon yield under drought conditions. We have studied a wide range of physiological responses that may be affected by drought conditions to compare and contrast the responses in *Miscanthus* with those in other more conventional crops. We have focussed on *M.* x *giganteus* because much agronomic research has been done on this sterile triploid hybrid species in Europe and the USA, and it is most widely grown for commercial purposes within the *Miscanthus* genus. Another reason for focussing on *M.* x *giganteus* is because drought has been predicted to have a strong negative impact on its performance in terms of biomass yield (Richter et al., 2008; Heaton et al., 2010).

The first observed physiological response in *M.* x *giganteus* under water stress conditions was a decrease in the rate of stem elongation. This stem elongation was significantly different between control and drought from day 20. This is consistent with many crop species in which growth inhibition during drought is primarily due to loss of turgor arising from lack of water availability (Farooq et al., 2009). Inhibition of stem elongation and leaf expansion reduces the demand for metabolites in the plant enabling the synthesis of protective compounds required for osmotic adjustment (Chaves et al., 2003). Leaf and stem elongation have been shown to be sensitive to changes in plant and soil water status in other species including maize (Hsiao et al., 1970; Westgate and Boyer, 1985). Sobrado (1986) reported a strong relationship between leaf expansion rate and predawn leaf turgor in tropical maize varieties; however the relative stem and leaf extension rate was barely associated with grain yield under stress. *Miscanthus* is cultivated for lignocellulosic biomass and all above ground biomass is harvested and stem traits (elongation and stem number) correlate strongly with yield (Robson et al., 2013). We therefore expected to see significant associations between, in particular, stem elongation rate under stress and yield in *Miscanthus*. Comparing day 24 and 32 there is little stem elongation and leaf expansion indicating that growth becomes negligible when the soil moisture content drops below 0.1 m^3^ m^3^, reflected by the dry above ground biomass remaining constant over this period.

Measures reflecting the plant water status (leaf RWC, above ground water content and fresh weight) showed a decrease on day 24, becoming significantly different from that of the controls by day 32. The dry weight of drought-treated plants remained the same between day 24 and 32. These results indicate a loss of water from above ground tissues under mild to moderate drought stress but not diminished biomass accumulation. The fact that the mean dry weight between control and treatment were the same on day 24 was somewhat surprising given that a significant reduction in stem elongation was observed in drought treated plants from day 20 onward. However, our data show that photosynthesis is rather resilient in *M.* x *giganteus* under drought stress (discussed below) not showing any significant impact on photosynthetic performance prior to day 24.

Photosynthesis is one of the key processes of primary metabolism and as such plays a major role in the plants response to low water stress conditions (Chaves et al., 2003). The photosynthetic process is affected by water deficits and the impact varies with intensity of the stress. At day 24 changes in photosynthetic measurements were seen with an increase in stomatal resistance. An initial moderate, but significant, increase was observed for 4 days when soil moisture dropped below 0.1 m^3^ m^3^(day 24–28) before rapid increases in resistance were observed under more severe drought conditions. Stomatal closure, caused by drought induced ABA synthesis, prevents water loss through transpiration. The stomatal conductance to water vapor decreases as the resistance increases. This leads to a decrease in intercellular carbon dioxide concentration and therefore inhibits photosynthesis. It has been previously shown that *M.* x *giganteus* shows little stomatal regulation under mild drought compared to *M. sinensis* which has shown more effective stomatal control under water limiting conditions (Clifton-Brown and Lewandowski, 2000). In addition to reduced CO_2_ diffusion through the stomata, water stress also results in reduced CO_2_ diffusion through the leaf mesophyll (Lawlor and Cornic, 2002). It was therefore expected that changes in chlorophyll content and fluorescence would be seen shortly after the changes to stomatal aperture. However, these two proxy measurements for photosynthetic performance were only affected toward the end of the experiment when drought was more severe, suggesting that *M.* x *giganteus* employs a drought tolerance strategy, i.e., it continued to function in spite of water stress indicating lack of drought adaptation, compared to the drought avoidance strategy previously seen in *M. sinensis*. *F*_v_/*F*_m_ is however not particularly sensitive to changes in photosynthetic capacity under drought (Percival and Sheriffs, 2002), and therefore may not be able to detect the initial decrease in photosynthesis, explaining the delay seen between increased stomatal closure and decrease in photosynthetic performance. The response seen in *M.* x *giganteus* is similar to the response seen in maize where dehydration tolerant genotypes were shown to maintain open stomata and active photosynthesis under mild drought conditions (Benesova et al., 2012). Unlike *F*_v_/*F*_m_ which utilizes only extreme values of chlorophyll fluorescence, the PI parameter is more comprehensive and incorporates multiple parameters including absorption and trapping of excitation energy, electron transport beyond the primary plastoquinone and dissipation of excitation energy. The PI parameter has been used in several studies of photosynthetic performance (Clark et al., 2000; Hermans et al., 2003; Strauss et al., 2006; Oukarroum et al., 2007). In this study PI was slightly more sensitive than the maximum quantum yield of PSII (*F*_v_/*F*_m_). The log linear correlation between PI and the quantum yield of electron transport suggests that changes in electron transport beyond Qa determined the changes in PI during drought treatment, a similar result was seen in drought studies of barley cultivars, mung bean (*Vigna radiata) *and Brassica (*Brassica juncea*; Misra et al., 2001; Oukarroum et al., 2007).

Biochemical tolerance responses of crops to drought have been linked to changes in the metabolic pathways leading to production of sugars, sugar alcohols, amino acids, and polyamines (reviewed in Seki et al., 2007). Therefore, metabolomics-based approaches are particularly appropriate when investigating plant responses to drought. In this work, we sought to demonstrate the validity of our metabolomics approach to investigate drought in *Miscanthus* rather than conduct an in depth characterization. However, to demonstrate the biological relevance of our study, we extracted *m/z *corresponding to the proline biosynthetic pathway, one of the largest sources of variation in our experiment, and sought to describe treatment difference based solely on these variables.

Drought induced accumulation of proline, caused by both activation of its biosynthesis and the inactivation of its degeneration, is considered to act as an osmoprotectant, a ROS scavenger, and a molecular chaperone stabilizing the structure of proteins, thereby protecting cells from damage caused by stress (Delauney and Verma, 1993; Hare and Cress, 1997; Szabados and Savoure, 2010). For example, overproduction of proline has been shown to result in increased tolerance to osmotic stress in transgenic plants (Kishor et al., 1995; Zhu et al., 1998; Yamada et al., 2005). Here we have shown that proline biosynthesis is a drought-affected metabolic trait in *M.* x *giganteus*. In our analyses, *m/z *linked to metabolites in the proline pathway allowed responses at day 24 to be distinguished between treatments (D1 and C1), thereby suggesting that biochemically relevant changes linked to drought were being measured in our experiment. It is generally accepted that under conditions of water deprivation or extreme salinity, proline accumulation serves as a defense against osmotic challenge by acting as a compatible solute (Hare and Cress, 1997). The metabolite analysis has shown that at D1, corresponding to mild-moderate drought conditions, the precursor for proline biosynthesis L-glutamate-semi-aldehyde and L-proline itself began to increase. Significantly, L-glutamate-semi-aldehyde and L-proline are respectively the products of two enzymes, pyrroline-5-carboxylate synthetase (P5CS), and pyrroline-5-carboxylate reductase (P5CR) which play major roles in the proline biosynthetic pathway (Delauney and Verma, 1993). Further increases in proline metabolite concentrations within the leaves confirm the importance of proline in the drought stress response of *Miscanthus*. This could be due to the function of increased proline as a molecular chaperone able to protect protein integrity and enhance the activities of different enzymes (Rajendrakumar et al., 1994). The enhanced rate of proline biosynthesis in chloroplasts can contribute to the stabilization of redox balance and maintenance of cellular homeostasis by dissipating the excess of reducing potential when electron transport is saturated during adverse conditions (Szabados and Savoure, 2010).

With *Miscanthus*, metabolomics could not only define the mechanisms of drought tolerance but also indicate biochemical markers for maximal biomass yield under drought to be exploited in germplasm selection/ breeding programes. This current work is the first application of metabolomics to investigate drought in *Miscanthus*. In applying these approaches, we demonstrated the use of appropriate sampling so that multivariate models describing the underlying biochemical changes could be defined. The biochemical changes detected by our metabolite profiling approach were sufficiently pronounced to give treatment specific separation by non-supervised PCA. These experimental data are currently being analyzed in great detail to identify further potential sources of drought tolerance.

**FIGURE 10:**
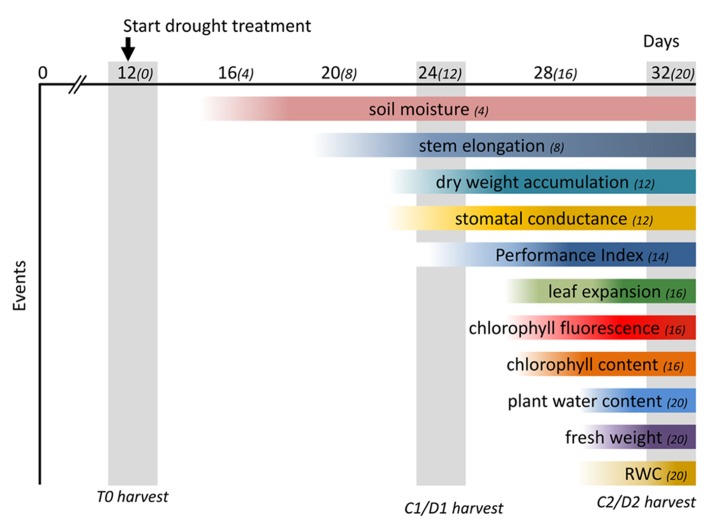
**Time-course of physiological changes observed after drought treatment. Soil moisture content decreased significantly 4 days after drought treatment (DADT).** The earliest physiological change observed was a decrease in stem elongation (8 DADT) followed by a significant decrease of stomatal conductance and dry weight accumulation (12 DADT). The multi-parametric performance index which incorporates the main photochemical processes was significantly affected at 14 DADT. Leaf expansion rate decreased significantly 16 DADT, although a declining trend could be observed from 8 DADT onward. A significant decline in chlorophyll fluorescence at 16 DADT was accompanied by a decrease of chlorophyll content. Finally, above ground biomass moisture content, fresh weight and RWC all declined significantly at 20 DADT. Numbers in parenthesis indicate days after start of drought treatment. The DADT indicated after each physiological event indicates the day that a significant effect was first observed. Gray vertical bars indicate days for destructive harvests.

The application of progressive drought enabled us to monitor and evaluate the physiological changes in *M.* x *giganteus* triggered over a period of 20 days after cessation of watering. A moderate drought treatment may allow plants to reach a new homeostasis with reduced growth (Harb et al., 2010) and a better understanding of this response will allow for the selection of plants that can tolerate limited water availability in temperate climates (Skirycz et al., 2011). *M.* x *giganteus* is currently grown over a range of geographies that are expected to experience more erratic climatological conditions including prolonged periods of drought. The progressive decrease in soil moisture content in drought-treated *M.* x *giganteus* plants allowed us to monitor the associated physiological changes throughout the experiment, summarized in **Figure 10**, providing valuable information on how *M.* x *giganteus* responds to drought stress. We have shown here that stem elongation is the first measure to be affected. This is therefore a good indicator of early or mild drought stress, with photosynthetic ability being affected under more severe stress. Stomatal conductance was one of the last physiological responses to be affected by drought stress in *Miscanthus*, this confirms a previous study that suggested *M.* x *giganteus* was poor at controlling stomatal aperture (Clifton-Brown and Lewandowski, 2000). Growth is one of the most drought-sensitive physiological processes with water-stress limiting growth more than any other abiotic stress (Shao et al., 2008)*.* The influence of water deficit and assimilate distribution depends on the stage of growth, with the most rapidly growing organ being most vulnerable to the stress (Nandwal et al., 1992). However the initial decline in elongation growth in drought-stressed *Miscanthus* was not associated with decreased biomass accumulation but resulted from a redistribution of resource allocation. Since all above ground biomass is harvested in *Miscanthus*, redistribution of resources among aerial parts of the plant will not affect yield and therefore despite this being the most dramatic and sensitive response it is unlikely to be a useful breeding target for mild drought conditions. However, a shift in assimilate partitioning between structural and non-structural carbohydrates could potentially impact on biomass quality and conversion for bioenergy and bioproducts. It is possible that resources are also redistributed to rhizomatous tissue which would affect yield and it will be interesting to follow resources in drought-stressed plants to determine the relative sensitivities of different resource allocations. Such detailed physiological data provides a platform for the future integration of physiological events with associated drought-induced metabolites and transcripts, enabling the identification of genes and pathways for the improvement of drought tolerance in *Miscanthus* through implementation in *Miscanthus *breeding programes and/or genetic engineering approaches.

## Conflict of Interest Statement

The authors declare that the research was conducted in the absence of any commercial or financial relationships that could be construed as a potential conflict of interest.
